# Change in the postoperative intervertebral space height and its impact on clinical and radiological outcomes after ACDF surgery using a zero-profile device: a single-Centre retrospective study of 138 cases

**DOI:** 10.1186/s12891-021-04432-0

**Published:** 2021-06-14

**Authors:** Haimiti Abudouaini, Chengyi Huang, Hao Liu, Ying Hong, Beiyu Wang, Chen Ding, Yang Meng, Tingkui Wu

**Affiliations:** 1 Department of Orthopedic Surgery, West China Hospital, Sichuan University, No. 37 Guo Xue Xiang Rd, Chengdu, 610041 Sichuan China; 2 Department of Anesthesia and Operation Center / West China School of Nursing, West China Hospital, Sichuan University, No. 37 Guo Xue Xiang, Chengdu, 610041 Sichuan China

**Keywords:** Anterior cervical decompression and fusion, Intervertebral height, Zero-profile, Fusion rate, Adjacent segment degeneration

## Abstract

**Background:**

The effects of postoperative intervertebral height (IH) changes on the clinical and radiological outcomes after anterior cervical decompression and fusion (ACDF) surgery using a zero-profile device remain unclear.

**Methods:**

We retrospectively reviewed patients who had undergone ACDF using a zero-profile device from March 2012 to February 2016 at our institution. Based on the postoperative IH variation, the patients were divided into group A with postoperative IH 0 to 2 mm, group B with postoperative IH 2 to 4 mm, and group C with postoperative IH greater than 4 mm. Clinical efficacy was evaluated using JOA, VAS, and NDI scores in the groups. Imaging parameters including the IH, cervical lordosis, fusion rate, intervertebral foramen (IVF) diameter and complications such as subsidence, dysphagia, and ASD were also compared across the three groups.

**Results:**

The average IH increased significantly from 6.72 mm preoperatively to 10.46 mm 1 week after surgery, and then gradually decreased to 7.48 mm at the final follow-up. The fusion rate was 61.90% in group A, 63.23% in group B, 53.57% in group C at 3 months, 73.81% in group A, 79.41% in group B, 67.86% in group C at 6 months, 90.48% in group A, 95.59% in group B, 92.86% in group C 1 year after surgery, and at the last follow-up, the fusion rate of three groups was all 100%. The IVF diameter was 6.52 ± 1.80 mm in group A, 9.55 ± 2.36 mm in group B, and 9.34 ± 1.62 mm in group C. ASD at the superior and inferior levels affected 11.90 and 16.67% patients in group A, 5.88 and 7.38% in group B, and 14.28 and 10.71% in group C. Regarding the 3 groups, the subsidence rates were 7.14, 4.41, and 14.29%, respectively.

**Conclusions:**

No clear correlation was found between IH changes and clinical efficacy within a year of surgery. However, the IH may affect various complications after ACDF. If postoperative IH changes are maintained at 2 to 4 mm after a year, a satisfactory imaging parameters and relatively low complications may be achieved after ACDF surgery using a zero-profile device.

## Background

Cervical disc degenerative disease (CDDD) is a spinal disorder that commonly affects middle-aged and older adults and may cause neck discomfort, radiating upper extremity pain, and neurologic abnormalities. Surgery is recommended if the patient does not respond to conservative treatment. Anterior cervical decompression and fusion (ACDF) using a traditional plate-cage construct (PCC) system is the main spinal surgery approach to treat symptomatic cervical disc disease [1]. However, postoperative axial pain and biomechanical instability leading to degeneration at the adjacent stage are likely caused by improper intervertebral distraction [2–5]. A zero-profile device is an alternative effective ACDF implant that can reduce adjacent segment degeneration to avoid implant contact with soft tissue in front of the cervical spine, likely preventing postoperative dysphagia [6–8].

Satisfactory reconstruction and consistent maintenance of the intervertebral height (IH) influence the cervical surgery outcome. IH distraction is associated with neck pain, the occurrence of ASD and neural functional recovery after cervical surgery [5]. However, compared with studies examining surgical skills such as IH distraction and reconstruction techniques, few studies have assessed the connection with IH maintenance and clinical efficacy after ACDF using a Zero-p device.

Here, we examined IH changes following zero-profile ACDF and analysed the relationship between IH and clinical efficacy and imaging parameters.

## Methods

Ethical approval for this study was granted by our institutional ethics committee. All the participants provided informed consent for analysis of their clinical data.

### Patient population

The data on single-level CDDD were collected retrospectively from March 2012 to January 2016 at our institution. The primary inclusion criteria were patients aged 18–65 years, symptomatic cervical disc degenerative diseases (CDDDs) with spondylotic radiculopathy or myelopathy at 1 level from C3 to C7 that correlate with imaging findings, patients showing a poor effect on conservative treatment or unclear improvement after at least 3 months with a worsening condition. Patients were excluded if they had received cervical disc arthroplasty (CDA), were treated with hybrid surgery (CDA incorporated with fusion), had undergone ACDF using another implant, or had been treated with multilevel surgery. Patients with infections, osteoporosis, spinal fractures, spinal deformity, allergy to the device material, ankylosing spondylitis, rheumatoid arthritis, and previous cervical spine surgery were also excluded.

### Surgical technique

All anterior fusions were performed using the Smith-Robinson technique and a right-sided approach by the same surgeon. After confirmation and exposure of the appropriate vertebral levels, a Caspar distracter was used, and disc material was removed. The endplate cartilage was scraped using a curette or high-speed electric drill to prepare for bone grafting. The posterior longitudinal ligament, osteophytes, and other compressive elements were removed to ensure adequate dural and neural decompression. After measuring the intervertebral height and width, the appropriate Zero-P implant filled with b-tricalcium phosphate was inserted with an implant holder/aiming device.

### Postoperative management

The patients wore a soft collar for 3 months after surgery, began functional exercise a day after the operation and were given a home exercise regimen at discharge.

### Clinical evaluation

The clinical outcomes were evaluated using the visual analog scale (VAS) arm and neck score, Japanese Orthopedic Association (JOA) score, and neck disability index (NDI). The VAS evaluated neck and arm pain, the JOA score evaluated myelopathy status, and the NDI assessed neck function. These clinical outcomes were measured preoperatively and at 3 months, 6 months, and 12 months and at the last postoperative follow-up.

### Radiologic assessment

**Fig. 1:**
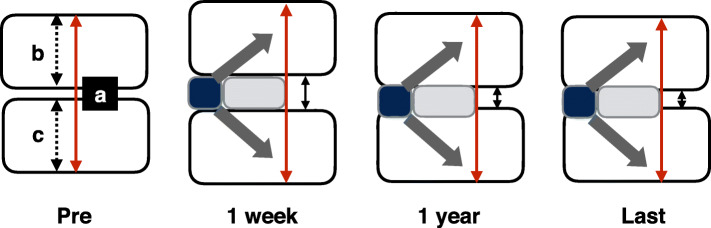
Representation of the radiographic measurement of the intervertebral height (IH) and its postoperative trend. The IH (black solid arrow between two vertebrae) = a - (b + c), where a (red arrow) is the distance from the midpoint of the upper endplate of the upper vertebral body and that of the lower endplate of the lower vertebral body, b is the distance from the midpoint of the upper endplate of the upper vertebral body to the midpoint of the lower endplate, and c is the distance from the midpoint of the upper endplate to the midpoint of the lower endplate

Independent radiologists performed radiographic imaging using standing lateral, flexion and extension radiographs. Radiologic measurements included the IH, cervical curvature, the functional spine unit (FSU), the intervertebral foramen (IVF) diameter, adjacent segment degeneration (ASD), the fusion rate and implant subsidence. The IH was calculated using the formula a-(b + c), where a is the distance from the midpoint of the upper endplate of the cephalad vertebral body to the lower endplate of the caudal vertebral body, b is the distance from the midpoint of the upper endplate of the cephalad vertebral body to the midpoint of the lower endplate, and c is the distance from the midpoint of the upper endplate to the midpoint of the lower endplate (Fig. 1). Cervical sagittal alignment was measured at the C2–7 angle. Local kyphosis was measured using the endplate method [9]. The FSU angle refers to the angle between lines drawn at the superior margin of the superior vertebral body and inferior margin of the inferior body. ASD is defined as the presence of 1) new or enlarged ossification of the anterior longitudinal ligament, 2) new or increased narrowing of the disc space by > 30%, 3) new or obvious enlarging osteophyte formation and 4) endplate sclerosis [10]. Radiological fusion refers to the presence of ≤2° motion and/or ≤ 2 mm of motion of the interspinous distance on flexion-extension X-rays [11]. Implant subsidence refers to a loss in the FSU height > 2 mm.

### Statistics

Radiographic assessments were performed twice by two independent surgeons, and the mean values were used for statistical analysis. The results are presented as means ± standard deviation. Student’s t-test or the Mann–Whitney U test was used to compare continuous data between two groups, depending on whether the data were normally distributed. Chi-squared test or Fisher’s exact test was used to compare categorical data between the groups. Odds ratios with 95% confidence intervals were calculated for differences between the groups. P = < 0.05 was considered statistically significant. SPSS version 22.0 (IBM Corp.) was used for statistical analyses.

## Results

One hundred forty-eight patients were enrolled in the study; among them, ten patients were excluded because of incomplete postoperative imaging data and did not attend the final follow-up. Thus, 138 consecutive patients (63 female and 75 male) qualified for further analyses. The mean age was 48.03 ± 9.51 years, with a mean follow-up of 22.83 ± 6.272 months (range: 18–36 months). Twelve patients were at the C3–4 level, 34 at the C4–5 level, 71 at C5–6, and 21 at C6–7.

### Intervertebral height

**Fig. 2:**
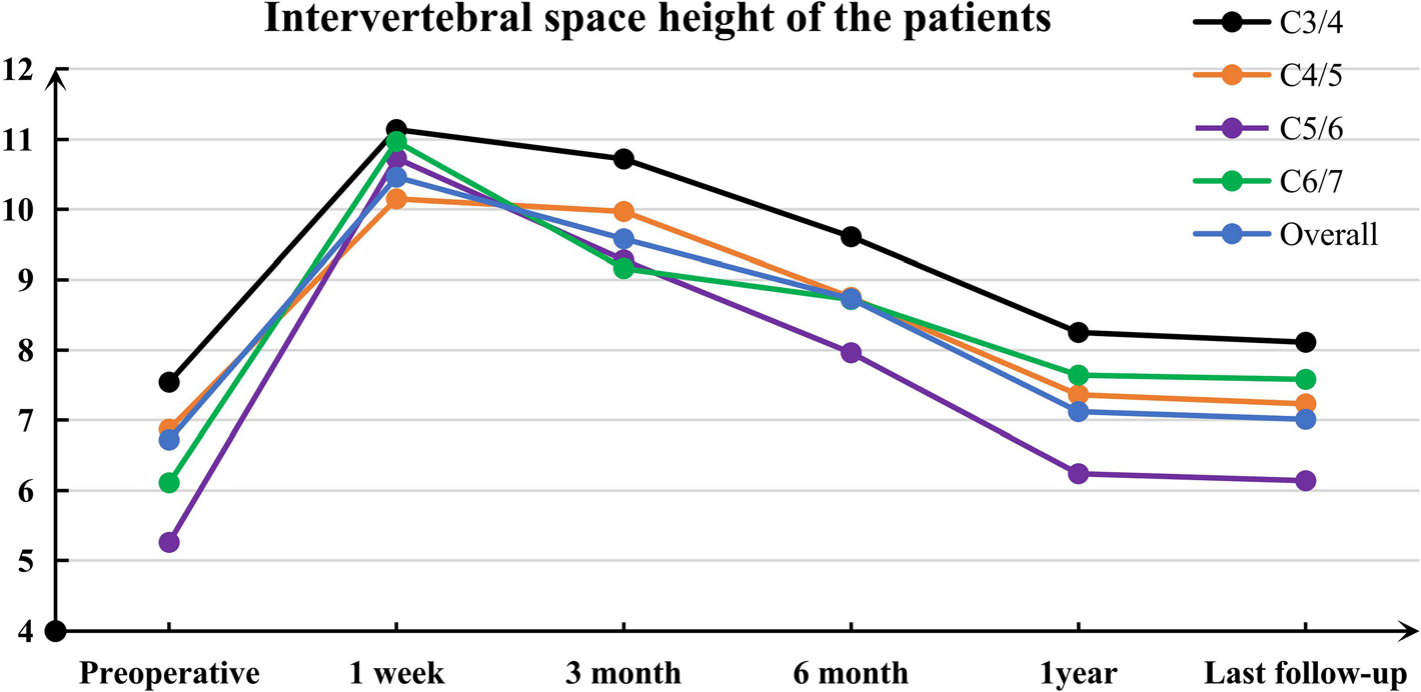
Trend chart of the intervertebral height (IH) after ACDF using the Zero-P device. The IH increased significantly 1 week after surgery, and then it was progressively reduced within the first year. However, a balance was achieved and no obvious reduction was observed in the IH 1 year after surgery

The average IH increased significantly from 6.72 mm preoperatively to 10.46 mm at 1 week after surgery. After that, the average IH progressively decreased to 9.58 mm at 3 months, 8.73 mm at 6 months, and 7.58 mm at 1 year (*p =* < 0.05). The average IH was 7.48 mm at the final follow-up (*p =* > 0.05) (Table 1, Fig. 2). To investigate the great importance of maintaining the IH after ACDF using a zero-profile device, the patients were grouped by postoperative IH change into three groups; the variation value of IH was 0–2 mm in group A, 2–4 mm in group B, and greater than 4 mm in group C. Group A comprised 42 patients (25 male and 17 female; average age = 45.06 ± 6.31 years), group B comprised 68 patients (40 male and 28 female; average age = 43.21 ± 7.53 years), and group C comprised 28 patients (16 male and 12 female; average age = 46.51 ± 7.6 years). The demographic factors, size of the zero-profile device and preoperative ASD were not significantly different among the 3 groups (Table 2).

**Table 1:** Intervertebral space height of the patients

	Preoperative	1 week	3 month	6 month	1 year	Last follow-up
C3/4	7.54	11.14	10.72	9.61	8.25	8.11
C4/5	6.87	10.15	9.97	8.75	7.36	7.23
C5/6	5.26	10.73	9.28	7.96	6.17	6.14
C6/7	6.11	10.97	9.16	8.72	7.64	7.58
Overall	6.72	10.46	9.58	8.73	7.58	7.48

**Table 2:** Demographic and baseline data

Group	Group A (ISH change < 2 mm)	Group B (ISH change 2-4 mm)	Group C (ISH change > 4 mm)	P
No.	42	68	28	
Age (y)	45.06 ± 6.31	43.21 ± 7.53	46.51 ± 7.61	0.061
Male	25	40	16	0.264
Female	17	28	12	0.162
Level (%)				
3/4	9.52	7.35	10.71	0.281
4/5	23.81	26.47	21.43	0.310
5/6	52.38	50.00	53.57	0.346
6/7	14.29	16.18	14.26	0.331
Implant height				
5 mm	7.14% (3/42)	8.82% (6/68)	10.71% (3/28)	0.873
6 mm	57.14% (24/42)	54.41% (37/68)	57.14% (17/28)	0.848
7 mm	30.95% (13/42)	30.88% (21/68)	28.57% (7/28)	0.830
8 mm	4.76% (2/42)	5.88% (4/68)	3.57% (1/28)	0.890
Preoperative ASD				
Superior	9.52% (4/42)	7.35% (5/68)	7.14% (2/28)	0.905
Inferior	14.28% (6/42)	11.76% (8/68)	10.71% (3/28)	0.888

*ISH* Intervertebral space height, *ASD* Adjacent segment degeneration

### Clinical outcomes

The clinical symptoms markedly improved after the operation in all the patients, and the mean JOA score was elevated in all the groups (Table 3). The mean VAS and NDI scores decreased significantly. No significant differences were found in the clinical parameters among the three groups.

**Table 3:** JOA, VAS and NDI scores for three groups

	ISH change< 2 mm	ISH change 2-4 mm	ISH change > 4 mm	P
JOA scores				
one week	11.73 ± 1.86	12.92 ± 2.46	12.84 ± 2.59	0.739
12-month	13.35 ± 2.23	13.66 ± 1.47	13.30 ± 1.62	0.642
VAS scores				
1 week	1.95 ± 0.58	1.91 ± 0.22	1.86 ± 0.64	0.530
1 year	1.82 ± 0.62	1.75 ± 0.84	1.69 ± 0.27	0.963
NDI scores				
one week	21.74 ± 0.68	21.67 ± 3.59	21.46 ± 2.94	0.744
12-month	19.53 ± 0.82	18.95 ± 4.67 ^a, b^	17.19 ± 4.22	0.528

### Radiological outcomes

The preoperative radiological parameters, postoperative FSU and cervical alignment were not significantly different among the three groups (all, *p =* > 0.05). The fusion rate was 61.90% in group A, 63.23% in group B, 53.57% in group C at 3 months, 73.81% in group A, 79.41% in group B, 67.86% in group C at 6 months, 90.48% in group A, 95.59% in group B, 92.86% in group C 1 year after surgery, and at the last follow-up, the fusion rate of three groups was all 100%. The fusion rate of patients in group C was significantly lower than that in the other two groups in the first 6 months (*p* = < 0.05). The mean IVF diameter was 6.52 ± 1.80 mm (variation = 2.58 ± 0.51 mm) in group A, 9.55 ± 2.36 mm (variation = 0.87 ± 1.21 mm) in group B, and 9.34 ± 1.62 mm (variation = 1.47 ± 1.30 mm) in group C. The IVF diameter in group A was significantly different than that in the other two groups, while the variation differed significantly between groups A and B 1 year post-surgery (Table 4).

**Table 4:** Radiographic assessments of patients in three groups

Group	ISH change< 2 mm	ISH change 2-4 mm	ISH change > 4 mm
C2–7 Cobb angle (°)			
1 week	14.52 ± 3.6	12.47 ± 2.9	13.65 ± 3.2
1 year	12.26 ± 9.36	11.91 ± 3.1	11.34 ± 4.5
Variation value	1.40 ± 4.80	1.15 ± 2.4	1.33 ± 2.07
Cobb angle of fused segments (°)			
1 week	16.4 ± 2.5	14.3 ± 2.9	16.1 ± 2.2
1 year	12.8 ± 1.7	12.4 ± 1.5	13.5 ± 1.0
Variation value	1.8 ± 3.2	2.1 ± 1.8	3.0 ± 2.9
Diameter of IVF (mm)			
1 week	9.14 ± 2.38	10.54 ± 1.89	10.83 ± 1.50
1 year	6.52 ± 1.80	9.55 ± 2.36^a^	9.34 ± 1.62^a^
Variation value	2.58 ± 0.51	0.87 ± 1.21^a^	1.47 ± 1.30
Fusion rate (%)			
3 months	61.90% (29/42) ^c^	63.23% (43/68) ^c^	53.57% (15/28)
6 months	73.81% (31/42) ^c^	79.41% (54/68) ^c^	67.86% (19/28)
1 year	90.48% (38/42)	95.59% (65/68) ^c^	92.86% (26/28)
Final follow-up	100%	100%	100%

*ISH* Intervertebral space height, *IVF* Intervertebral foramen; Diameter of IVF = (longitudinal diameter of IVF + transverse diameter of IVF) / 2

Based on radiography, the ASD at the superior level affected 11.90% (5/42) of group A patients, 5.88% (4/68) of group B patients, and 14.28% (4/28) of group C patients. The ASD at the inferior level affected 16.67% (7/42) of group A patients, 7.35% (5/68) of group B patients, and 10.71% (3/28) of group C patients. Group B rates were significantly lower than those of the other groups (*p* = < 0.05). The incidence of implant subsidence was 7.14% in group A, 4.41% in group B, and 14.29% in group C. The rates in group B were significantly different from those in group C for implant subsidence. The incidence rates of dysphagia and local kyphosis did not differ significantly among the groups (Table 5).

**Table 5:** Comparison of complications among three groups

Group	ISH change< 2 mm	ISH change 2-4 mm	ISH change > 4 mm
Adjacent-level degeneration			
Superior	28.57% (12)	13.24% (9)^a, c^	21.43% (6)
Inferior	30.95% (13)	16.18% (11)^a, c^	28.57% (8)
Axial symptoms	26.19% (11)	11.76% (8)^a^	17.85% (5)
Dysphagia	5.4% (2)	4.41% (3)	5.26% (1)
Local kyphosis	9.52% (4)	13.24% (9)	14.29% (4)
Implant subsidence	7.14% (3)	4.41% (3)^b^	14.29% (4)
Facet joint degeneration [11]	3 points	2 points	2 points

## Discussion

Effective IH improvement is crucial for good outcomes after cervical spine surgery. Undesirable postoperative IH has been linked to a higher incidence of postsurgery axial symptoms and ASD [12]. Thus, effective intraoperative restoration and postoperative maintenance of IHs are necessary. However, compared with surgical skills such as IH distraction and reconstruction techniques, few studies have assessed the association between IH maintenance and the clinical efficacy after ACDF using a Zero-p device.

The intervertebral height (IH) was effectively improved in all patients. The average IH rose significantly from 6.72 mm preoperatively to 10.46 mm 1 week before being progressively reduced to 9.58 mm at 3 months, 8.73 mm at 6 months, 7.58 mm at 1 year, and 7.50 mm at the last follow-up. Thus, the IH changed subtly 1 year after ACDF (Fig. 2). Our data revealed no clear association between the postoperative disc height change and any clinical outcomes in the 1st year after ACDF surgery.

**Fig. 3:**
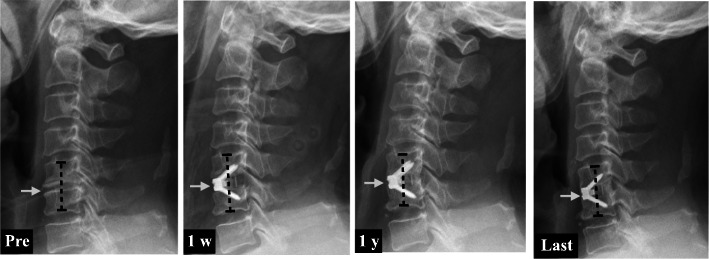
Case report: A middle-aged patient who had undergone C5/6 ACDF using the Zero-P device. The mean IH was increased significantly from 6.52 mm preoperatively to 10.37 mm at 1 week, and then it decreased to 7.84 mm at 1 year and 7.39 mm at the last follow-up (white arrows)

A study involving 37 1-level procedures, 50 2-level procedures and 13 3-level procedures evaluated the effects of the IH on overall outcomes after ACDF and found that the IH changed from a preoperative mean of 5.49 ± 1.17 mm to 6.62 ± 1.12 mm at 12 months postsurgery (mean change = 1.13 ± 1.33 mm) [13] (Fig. 3). Here, to avoid biomechanical changes due to adjacent surgical segments that may affect postoperative IH, we only included 1-level ACDF surgery with zero-profile implants.

Achieving complete nerve root decompression in cervical spondylotic radiculopathy patients with intervertebral foramen stenosis is challenging. Frequently, no remission for radicular pain or reoccurrence is observed after temporary relief in such patients. Although the pathogenesis of cervical radiculopathy is not completely understood, stenosis of the intervertebral foramina is considered among its main mechanistic underpinnings [14]. Additionally, narrowing of the intervertebral foramen after surgery is a risk factor for postoperative recurrence of neurological symptoms [15]. Therefore, the intervertebral foramen, as the doorway of the nerve root, plays an important role in radiculopathy and surgical treatment of intervertebral foramen diseases. Here, although the IVF diameter in group A patients was significantly smaller than that in groups B and C 1 year after surgery, this condition was not reflected in the patients’ radicular symptoms. Our study sought to verify the connection between IH variation and overall outcomes, reflecting the 1-year follow-up after surgery.

An in vitro biomechanical study used a calibrated distractor and a subminiature load cell on 17 cadaveric cervical specimens to investigate the effects of IH and distractive forces on a cervical spine model. In that study, the longer was the intervertebral space distance, the greater was the compressive load produced between the implant and vertebral body end plate, which may decrease the fusion rate [16]. Here, the fusion rate in group C patients was significantly lower than that in groups A and B in the first 6 months, possibly because the height of the intervertebral space declined too much in a short time. This effect may concentrate the compressive load in the endplate with the narrowest intervertebral space, negatively affecting bone fusion. This possibility should be tested by analysing the influence of stress distribution on bone fusion using ACDF models with different intervertebral heights through finite elements or biomechanical tests.

Prosthesis subsidence affects 7–25% of patients undergoing ACDF surgery using the Zero-P device [17–19]. Our data revealed 14.29% subsidence in group C patients, a level that was significantly higher than that in the other 2 groups. Because previous studies have identified various causes and risk factors for implant subsidence after ACDF [19, 20], it is unlikely that implant subsidence was caused by a single factor. However, our data clearly show that the lower fusion rate caused by an IH change > 4 mm is a non-negligible factor for the higher subsidence rate after ACDF.

We found that IH is strongly associated with the occurrence of ASD, consistent with a prior study [4]. In that study, the average IH variation was 1.8 mm in the ASD group vs 2 mm in the non-ASD group over a two-year follow-up. Li et al. [21] reported that excessive disc space distraction is a considerable risk factor for the development of radiographic ASD after patients had undergone ACDF polyetheretherketone (PEEK) cages with an anterior plate. The main reason may be that distraction of the fusion level by cage insertion exerts significant mechanical stress on the adjacent levels. Although prostheses with different design concepts were used, the same results were obtained in our study; ASD was significantly lower in group B than in groups A and C. Appropriate IH provides a better surgical view during decompression and prosthesis insertion, which is a prerequisite to improve the effectiveness and safety of using the Zero-p implant system. Small IHs might result in inadequate decompression or the formation of pseudarthrosis; conversely, large IHs may elevate mechanical stress on adjacent levels [16].

Our study has the following limitations. Because it was a retrospective study, patient selection bias was unavoidable. Thus, randomized controlled studies are needed to validate our findings. Although our measuring method was conducted according to previous studies, we acknowledge that potentially inherent radiographic imaging error may be a major limitation. Additionally, we did not evaluate effective methods for maintaining the postoperative IH. Given that our study mainly investigated the effects of postoperative IH changes on clinical and radiographic outcomes, further studies must focus on effective interventions for maintaining the postoperative IH at 2 to 4 mm.

## Conclusion

We found that ACDF surgery using a zero-profile device could not sustain the IH attained 1 week after surgery. No clear correlation was found between IH changes and clinical efficacy within a year of surgery. However, the IH may affect various complications within a year after ACDF. If postoperative IH changes can be maintained at 2 to 4 mm after a year, a satisfactory fusion rate and IVF diameter and a relatively low implant subsidence and ASD may be achieved after ACDF surgery using a zero-profile device.

## Data Availability

The datasets used and/or analysed during the current study are available from the corresponding author on reasonable request.

## Abbreviations

IH: Intervertebral height
ACDF: Anterior cervical decompression and fusion
JOA: Japanese Orthopedics Association
NDI: Neck Disability Index
VAS: Visual analogue scale
IVF: Intervertebral foramen
ASD: Adjacent segment degeneration
CDDD: Cervical disc degenerative disease
PCC: Plate-cage construct
CDA: Cervical disc arthroplasty
FSU: Functional spine unit
